# Sm_0.5_Sr_0.5_Co_1−x_Ni_x_O_3−δ_—A Novel Bifunctional Electrocatalyst for Oxygen Reduction/Evolution Reactions

**DOI:** 10.3390/molecules27041263

**Published:** 2022-02-14

**Authors:** Xingmei Liu, Yuwei Wang, Liquan Fan, Weichao Zhang, Weiyan Cao, Xianxin Han, Xijun Liu, Hongge Jia

**Affiliations:** Heilongjiang Provincial Key Laboratory of Polymeric Composite Materials, College of Materials Science and Engineering, Qiqihar University, Qiqihar 161006, China; lxm19980112@163.com (X.L.); zhang980502@163.com (W.Z.); weiyan19970606@163.com (W.C.); 03498@qqhru.edu.cn (X.H.); liuxijun2002@163.com (X.L.); jiahongge@qqhru.edu.cn (H.J.)

**Keywords:** Sm_0.5_Sr_0.5_Co_1−x_Ni_x_O_3−__δ_, perovskite, cathode electrocatalyst, OER/ORR

## Abstract

The development of non-precious metal catalysts with excellent bifunctional activities is significant for air–metal batteries. ABO_3_-type perovskite oxides can improve their catalytic activity and electronic conductivity by doping transition metal elements at B sites. Here, we develop a novel Sm_0.5_Sr_0.5_Co_1−x_Ni_x_O_3−δ_ (SSCN) nanofiber-structured electrocatalyst. In 0.1 M KOH electrolyte solution, Sm_0.5_Sr_0.5_Co_0.8_Ni_0.2_O_3−δ_ (SSCN82) with the optimal Co: Ni molar ratio exhibits good electrocatalytic activity for OER/ORR, affording a low onset potential of 1.39 V, a slight Tafel slope of 123.8 mV dec^−1^, and a current density of 6.01 mA cm^−2^ at 1.8 V, and the ORR reaction process was four-electron reaction pathway. Combining the morphological characteristic of SSCN nanofibers with the synergistic effect of cobalt and nickel with a suitable molar ratio is beneficial to improving the catalytic activity of SSCN perovskite oxides. SSCN82 exhibits good bi-functional catalytic performance and electrochemical double-layer capacitance.

## 1. Introduction

Due to the excessive use of fossil fuels, environmental pollution and energy shortages have become significant challenges for human survival. Therefore, it is urgent to develop novel and efficient energy conversion devices [1,2]. Natural energy sources such as wind and solar power are abundant, but their power output is subject to climate constraints [3]. Metal–air batteries have a good application prospect among many energy storage devices due to their advantages of low price, friendly environment, and good stability [4,5]. The cathode of the metal–air battery is open, and oxygen in the air can be continuously input. There are two critical electrochemical reactions at the cathode, namely oxygen reduction reaction (ORR) and oxygen evolution reaction (OER), which can determine the performance of the entire battery. Therefore, the selection of cathode catalysts is crucial. Up to now, precious metals such as Pt, IrO_2_, and RuO_2_ are still the most efficiently employed catalysts, but their high price and scarce content in the earth’s crust also limit their practical application [6,7,8]. Thus, it is imperative to develop non-noble metal bifunctional catalysts with good performance [9].

For ABO_3_-type perovskite oxides, the A-site is alkaline earth metals or rare earth metals with 12-fold oxygen. The B-site is a transition metal coordinated with six-fold oxygen located at the center of the octahedron. The electrochemical properties of ABO_3_ perovskite oxides are significantly improved by doping the A and/or B sites, making ABO_3_ one of the best candidates for non-noble metal catalysts [10,11,12]. Many perovskite oxides show good electrocatalytic activity. Sm_0.5_Sr_0.5_CoO_3−δ_ (SSC) on Vulcan XC-72R exhibited excellent bifunctional electrocatalytic performance for metal–air batteries [13]. La_0.5_Sr_0.5_NiMnRu_0.5_O_6_ (LSNMR) showed outstanding bifunctional ORR/OER activities. The ORR onset potential (E_onset_) of LSNMR was 0.94 V, which can be the perovskite with the best ORR activity in alkaline solution so far. The OER potential was 1.66 V at 10 mA cm^−2^ [14]. Ba_0.5_Sr_0.5_Co_0.8_Fe_0.2_O_3__−__δ_ (BSCF) remained highly stable for OER after 1000 cycles. For BSCF9002N2, the Tafel slopes of OER and ORR are 143 and 128 mV dec^−1^, respectively [15]. Sm_0.5_Sr_0.5_CoO_3−δ_ hollow nanofibers were hybridized with N-doped graphene to obtain a remarkable ORR/OER bifunctional catalyst in alkaline media [16]. Catalyst materials with various morphology and catalytic activity can be prepared by different preparation methods. Heteroatoms doping is an effective method to enhance the electrocatalytic activity of the catalysts [17].

Our previous studies [18,19] have shown that one-dimensional nanofiber-based Sm_0.5_Sr_0.5_CoO_3__−δ_ (SSC) exhibits excellent electrochemical performance. The doping of B-site in ABO_3_-type SSC perovskites can efficiently promote the electrocatalytic activity of the catalyst. The replacement between the two variant Ni and Co atoms in catalyst materials can generate the ligand effect, resulting in the acceleration of the charge transfer. The synergistic coupling effect of Ni^2+^ and Co^2+^ ions can afford bifunctional synergism and promote catalytic activity [20]. To the authors’ knowledge, the ORR/OER activities of Ni-doped Sm_0.5_Sr_0.5_CoO_3−δ_ at B-site have rarely been reported. In the present work, a novel nanofiber-structured Sm_0.5_Sr_0.5_Co_1−x_Ni_x_O_3−δ_ was synthesized by electrospinning, and the OER and ORR catalytic activities were explored.

## 2. Results and Discussion

**Figure 1:**
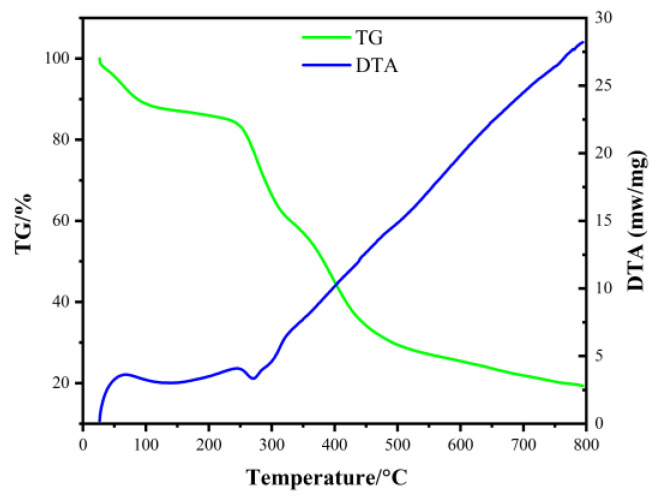
TG–DTA curves of the SSCN precursor with a scanning rate of 5 °C min^−1^.

To ascertain the optimal molar ratio of Co: Ni in SSCN catalysts, SSCN82, SSCN64, SSCN55, SSCN46, and SSCN28 were prepared by electrospinning and subsequent calcination. Figure 1 shows the typical TG–DTA curve of the SSCN precursor. The thermogravimetric process of the SSCN precursor can be divided into three stages: the weight loss in the range of 0~240 °C is due to the evaporation of water in the precursor, and the weight is reduced by 13.6% [21]. The weight loss between 244 °C and 401 °C is 39.7%, because metal nitrate will decompose within this temperature range [22]. At the same time, an apparent exothermic peak of metal nitrate can be found near 265 °C. The remaining 20.57% metal nitrate is further decomposed slowly at 406~654 °C. There was no apparent phase transformation after 654 °C, indicating that the SSCN precursor had been decomposed. To ensure the complete decomposition of the precursor, the SSCN nanofibers obtained by electrostatic spinning were calcined at 800 °C.

**Figure 2:**
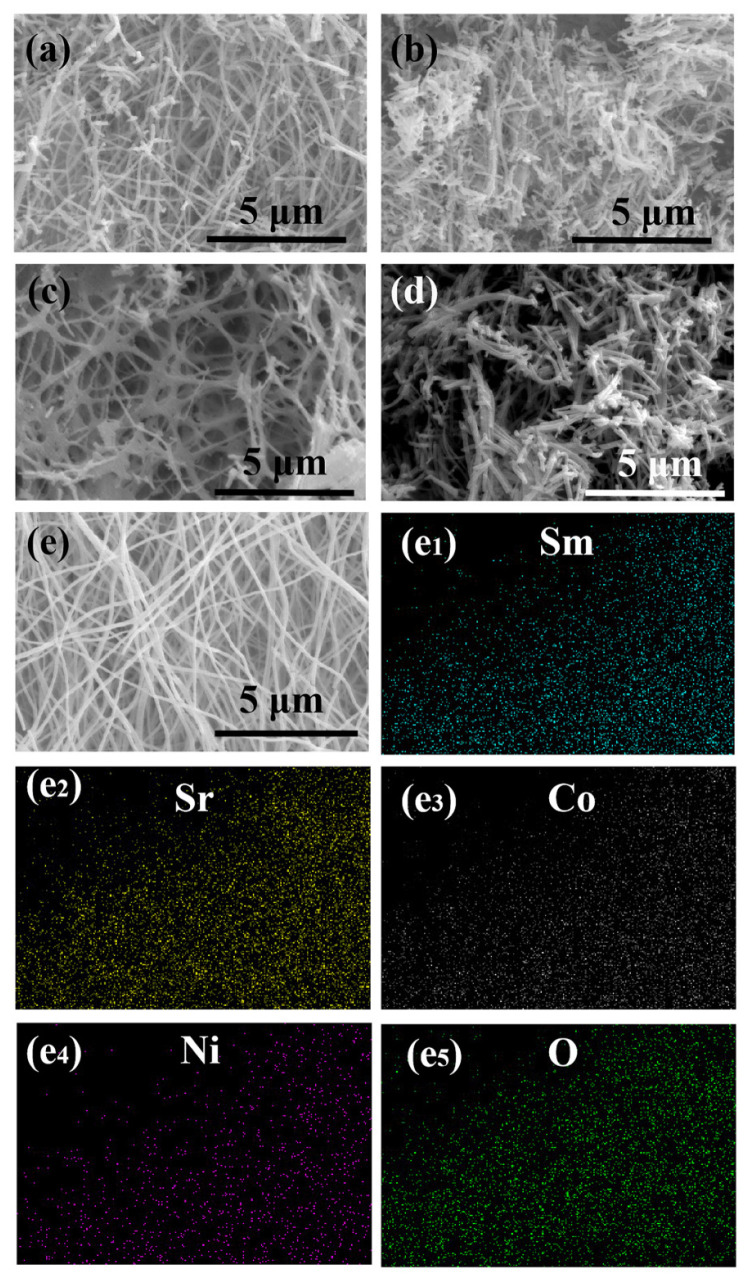
SEM images of (**a**) SSCN28, (**b**) SSCN46, (**c**) SSCN55, (**d**) SSCN64, and (**e**) SSCN82. (**e1**–**e5**) EDS element mappings of Figure 2e.

Figure 2a–e shows the SEM images of SSCN catalysts with different Co: Ni molar ratios after calcination at 800 °C for 2 h in air. It can be seen that the microstructure of SSCN fibers with different Co: Ni ratios is not the same. In comparison, the more Ni content in SSCN, the less likely it is to form a fiber structure, which may be due to the agglomeration of nickel in the calcination process. However, with the increase of Co content, SSCN82 nanofibers are not easy to fracture, and the diameter is relatively uniform. According to the literature report [16] and our previous research work [18,19], Sm_0.5_Sr_0.5_CoO_3−__δ_ (SSC) materials prepared by electrospinning have a good nanofiber structure. While for SSCN nanofibers, due to the intrinsic characteristics of easy agglomeration of Ni, the more nickel content in Sm_0.5_Sr_0.5_Co_1−x_Ni_x_O_3−__δ_, the more likely the fiber will fracture or even agglomerate. The increase in Co content is beneficial to the formation of SSCN long fibers [23,24]. Thus, uniform nanofibers without obvious breakage are presented in SSCN82 with the highest Co content. Figure e1–e5 shows the EDX spectra of SSCN82 nanofibers. It clearly shows the uniform spatial distribution of Sm, Sr, Co, Ni, and O elements in the SSCN82 catalyst. In particular, the distribution of nickel can fully prove that nickel has been evenly doped into samarium strontium cobalt oxide.

**Figure 3:**
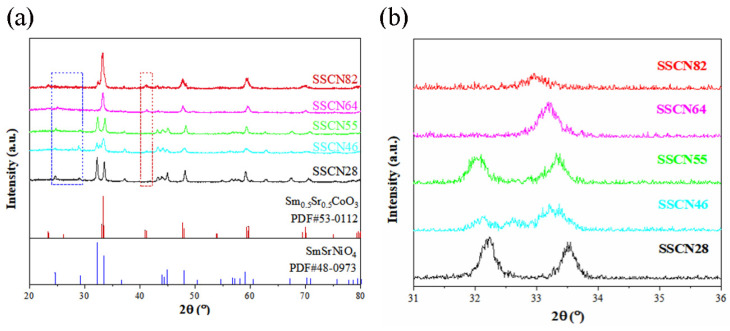
Wide-angle XRD patterns of SSCN82, SSCN64, SSCN55, SSCN46, and SSCN28 catalysts after calcination at 800 °C for 2 h. Standard X-ray diffraction peaks of SmSrCoO_3_ (PDF#53-0112) and SmSrNiO_4_ (PDF#48-0973) are given as guides to the eyes at the bottom. (**b**) A partial enlarged drawing of (**a**).

The wide-angle XRD patterns of the SSCN catalysts are shown in Figure 3a. The standard diffraction X-ray peaks of SmSrCoO_3_ (PDF#53-0112) and SmSrNiO_4_ (PDF#48-0973) are also given as guides to the eyes at the bottom of Figure 3a. All the diffraction peaks of SSCN were indexed to SmSrCoO_3_ and SmSrNiO_4_. The characteristic peaks appearing in Sm_0.5_Sr_0.5_Co_1__−__x_Ni_x_O_3__−__δ_ are consistent with the XRD pattern reported by Baek et al. [25]. The result indicates that the SSCN catalysts have a high crystalline characteristic after calcination at 800 °C. It is noteworthy that the peaks at about 2θ≈24.6°, 29.2°, 32.2°, and 33.4° are consistent with (011), (004), (013), and (110) for SmSrNiO_4_ (PDF#48-0973), respectively. The characteristic peaks increase with the increase of Ni content for Sm_0.5_Sr_0.5_Co_1__−__x_Ni_x_O_3__−__δ_. Peaks at about 2θ≈33.3°, 40.9°, and 41.2° can be well indexed to (121), (220), and (022) of SmSrCoO_3_ (PDF#53-0112), respectively. With the decrease of Co content, the characteristic peak gradually weakens until SSCN28, which cannot be detected due to too little Co content. That is also why SSCN82 and SSCN64 have one peak while SSCN55, SSCN46, and SSCN28 have two peaks in the range of 30° and 35° (see Figure 3b). Moreover, from Figure 3b, the diffraction peaks of SSCN shifted towards a larger angle with the decrease of the Co/Ni ratio. Because Co ions are replaced by larger Ni ions, resulting in the lattice expansion of the SSCN unit cell [26,27,28].

**Figure 4:**
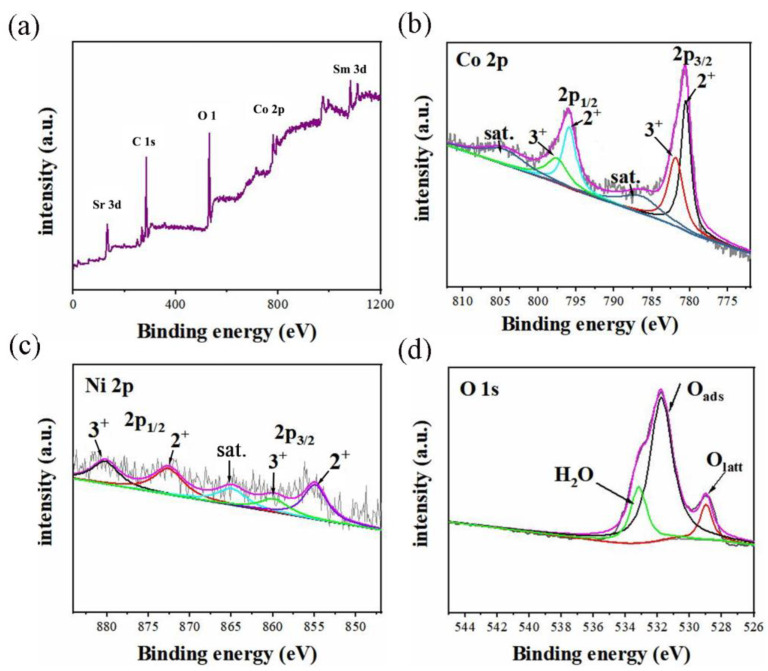
(**a**) XPS spectra of SSCN82 catalyst. High-resolution XPS spectra of (**b**) Co 2p, (**c**) Ni 2p, and (**d**) O 1s.

To further examine the surface composition and valence states of Co and Ni in SSCN, X-ray photoelectron spectroscopy (XPS) measurements were performed. The wide-scan spectrum of SSCN82 reveals the presence of Ni, Co, O, and Sm and Sr elements (Figure 4a). The Co 2p spectrum in Figure 4b shows the peaks of Co 2p_3/2_ and Co 2p_1/2_ along with their satellite peaks. For SSCN82, two peaks appear at 780.5 and 781.3 eV, which belong to Co 2p_3/2_ and indicate the presence of Co^2+^ and Co^3^^+^, respectively [21]. The Ni 2p spectrum (Figure 4c) shows 2p_3/2_ and 2p_1/2_ doublets due to spin–orbit coupling. The Ni 2p spectra of SSCN82 show two peaks at 853.8 and 859.3 eV corresponding to Ni 2p_3/2_ and a satellite peak at higher binding energies [29,30]. From Figure 4d, the O 1s peaks for the sample are located at 529, 531.7, and 533.1 eV, respectively, corresponding to lattice oxygen, absorbed oxygen, and absorbed water [31,32]. The binding energies of Ni 2p and Co 2p in SSCN82 reveal that the Ni and Co atoms are uniformly distributed in the crystal structure. It further demonstrated the formation of SSCN82, which was in agreement with XRD results.

For Co oxide-based catalysts, it has been found that the number of oxidation states of Co present in the catalyst is combined with its activity for the ORR and OER. The presence of Co^3+^ is associated with higher activity towards the OER, while Co^2+^ shows higher activity towards the ORR [33]. The variability of the valence states of the cobalt ions between Co^2+^ and Co^3+^ benefits attaining excellent bifunctional ORR/OER electrocatalysis. Similar characteristics of bifunctional activity were also observed for the other transition metal oxides [34]. Ni can undergo more than one oxidation–reduction reaction during the OER [33]. The effect of Ni content on the formation of a bimetallic Co-Ni oxide electrocatalyst was explored [35]. In general, adding Ni to the catalyst can improve the half-wave ORR potential and current densities, respectively.

**Figure 5:**
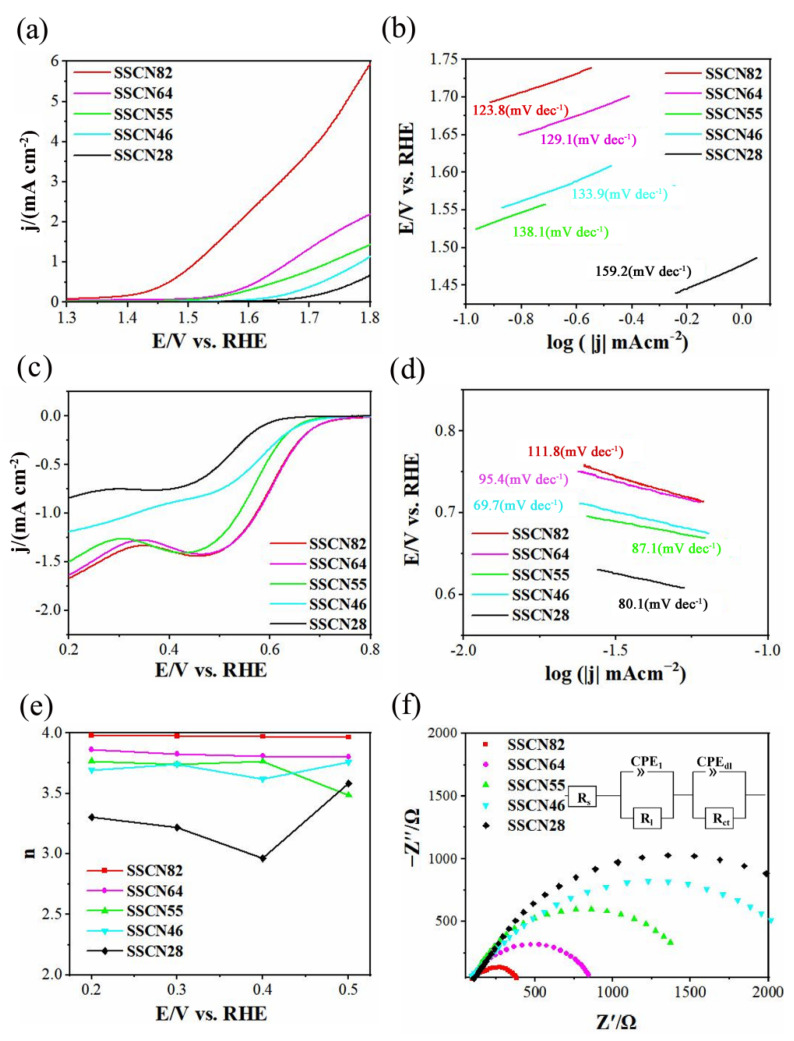
OER and ORR activities of SSCN. (**a**) OER polarization curves of SSCN28, SSCN64, SSCN55, SSCN46, and SSCN82 catalysts obtained in 0.1 M KOH solution with a scan rate of 5 mV s^−1^, and (**b**) the corresponding Tafel plots of SSCN catalysts. (**c**) ORR polarization curves of SSCN28, SSCN46, SSCN55, SSCN64, and SSCN82 obtained in O_2_-saturated 0.1 M KOH solution at 1600 rpm, and (**d**) the corresponding Tafel plots of SSCN catalysts. (**e**) Electron-transfer number (*n*) of SSCN catalysts. (**f**) Electrochemical impedance spectra at 1.664 V vs. RHE.

To evaluate the optimal molar ratio of cobalt and nickel of Sm_0.5_Sr_0.5_Co_1−x_Ni_x_O_3−__δ_, the OER and ORR activities were tested at a speed of 1600 rpm in 0.1 M KOH solution. Figure 5a shows the OER polarization curves of the SSCN catalysts. SSCN82 catalyst shows a higher current density at the same potential. The onset potential (*E*_onset_) of SSCN82 was 1.39 V, which was lower than the others. The results showed that SSCN82 had higher OER catalytic activity than the others. Figure 5b shows the Tafel slope obtained from the LSV curves. The OER Tafel slopes of SSCN82, SSCN64, SSCN55, SSCN46, and SSCN28 are 108.3, 137.7, 173.3, 170.4, and 180.9 mV dec^−1^, respectively. Among the five samples, the Tafel slope of SSCN82 is the lowest, which proves that it has faster OER reaction kinetics. SSCN82 generated a current density of 6.01 mA cm^−2^ at 1.8 V. For the sake of comparison, the results in our work and the values reported in literature [15,16,36,37,38,39] are summarized in Table 1. The OER performance of SSCN82 is comparable to the perovskite catalysts.

Furthermore, the ORR activities were also examined to demonstrate the bifunctionality of SSCN catalysts. Figure 5c shows the ORR polarization curves of SSCN at 1600 rpm. The Tafel slope (b) can be calculated by the equation: E = a + b log |j|. The Tafel plots of SSCN82, SSCN64, SSCN55, SSCN46, and SSCN28 are 111.8, 95.4, 69.7 87.1, and 80.1 mV dec^−1^, respectively. As shown in Figure 5e, the electron-transfer number of SSCN82 was closest to 4. It indicates that SSCN82 can restore O_2_ to OH^−^ via the desired four-electron pathway [16,40]. Combining the OER and ORR test results, it is obvious that SSCN82 has the most excellent OER and ORR performance, which proved that the synergy of Co and Ni with the suitable molar ratio is beneficial for the bifunctional activities [20]. In addition, electrochemical impedance spectroscopy (EIS) analysis can further study the catalytic kinetics of SSCN. The kinetic activity of different catalysts can be expressed by their charge transfer resistance (*R*_ct_). A lower *R*_ct_ means a faster kinetic reaction. Impedance spectra of the SSCN catalysts appeared as two capacitive arcs (Figure 5f), which can be fitted by the equivalent circuit (inset of Figure 5f). Here, *R*_s_ is the solution resistance, and CPE_1_ and CPE_dl_ are two constant phase elements. R_1_ represents the electron transport resistance of catalyst and electrode, and *R*_c__t_ the interfacial charge transfer resistance. SSCN82 shows the lowest *R*_ct_, indicating SSCN82 has the optimal electron and charge transport capability. The ORR/OER and CV test results divulge that a 0.8:0.2 molar proportion of Co: Ni is optimum for Sm_0.5_Sr_0.5_Co_1−x_Ni_x_O_3−__δ_. Combining the best morphological characteristic of SSCN82 nanofibers with the synergistic effect of cobalt and nickel with the optimal molar ratio is beneficial to improving the catalytic activity of SSCN82 [20].

**Figure 6:**
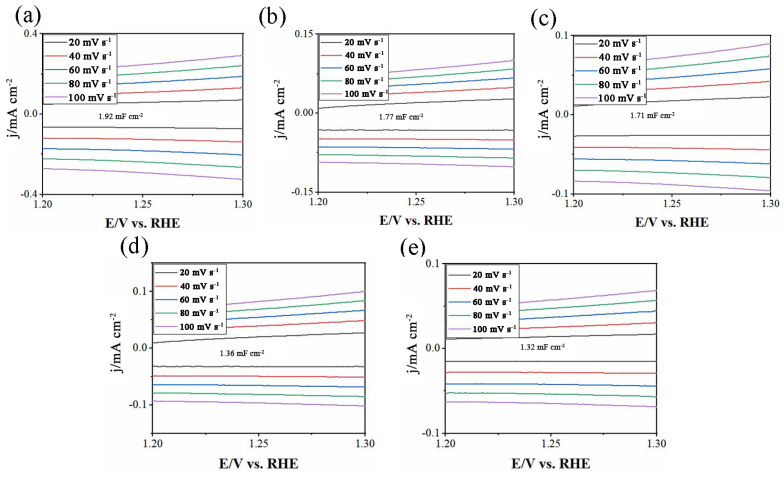
CV curves of (**a**) SSCN82, (**b**) SSCN64, (**c**) SSCN55, (**d**) SSCN46, and (**e**) SSCN28 catalysts obtained with different scan rates (20~100 mV s^−1^) between a potential range of 1.20~1.30 V vs. RHE.

For oxygen electrode catalysts, the electrochemical double-layer capacitance (*C*_dl_) is proportional to the active area of the catalyst [23], and the actual electrochemical active area can be calculated from CV test. Figure 6 shows the CV curves of SSCN catalysts with different cobalt–nickel ratios in the potential range of 1.20~1.30 V and at different scanning speeds (20~100 mV s^−1^). In the potential range, the transient non-Faraday current is only caused by the structural changes of the double electric layer. Therefore, the actual surface area of the electrode can be measured by studying the adsorption and desorption behavior of the electrode surface through the non-Faraday current in this range. It can be seen from the figure that the charge and discharge current of the double layer increases linearly with the increase of scanning speed. The *C*_dl_ values of SSCN82, SSCN64, SSCN55, SSCN46, and SSCN28 catalysts were 1.92, 1.77, 1.71, 1.36, and 1.32 mF cm^−2^, respectively. SSCN82 has the highest *C*_dl_ value and reactivity area, so it has the best catalytic activity among all SSCN catalysts.

## 3. Materials and Methods

Sm_0.5_Sr_0.5_Co_1−x_Ni_x_O_3−__δ_ (SSCN, x = 0.2, 0.4, 0.5, 0.6, and 0.8) nanofibers were synthesized by the electrospinning method. Stoichiometric amounts of samarium nitrate (Sm(NO_3_)_3_·6H_2_O), strontium nitrate (Sr(NO_3_)_2_), cobalt nitrate hexahydrate (Co(NO_3_)_2_·6H_2_O), and nickel nitrate (Ni(NO_3_)_2_·6H_2_O) with the molar ratio of 0.5:0.5:1-x:x were added into N, N-dimethyl formamide (DMF) under stirring. Then, polyvinylpyrrolidone (PVP) was dissolved into the above solution under constant stirring for several hours to form a clear and homogeneous electrospinning precursor solution. The electrospinning method was applied to synthesize the SSCN precursor nanofibers. Then the precursors were dried in a vacuum drying chamber at 150 °C for 4 h to remove the excess solvent, and subsequently calcined at 800 °C for 2 h in air with a rate of 3 °C min^−1^, obtaining the Sm_0.5_Sr_0.5_Co_0.8_Ni_0.2_O_3−__δ_, Sm_0.5_Sr_0.5_Co_0.6_Ni_0.4_O_3−__δ_, Sm_0.5_Sr_0.5_Co_0.5_Ni_0.5_O_3−__δ_, Sm_0.5_Sr_0.5_Co_0.4_Ni_0.6_O_3−__δ_, and Sm_0.5_Sr_0.5_Co_0.2_Ni_0.8_O_3−__δ_ samples. Accordingly, the samples were denoted as SSCN82, SSCN64, SSCN55, SSCN46, and SSCN28, respectively.

Thermal gravimetric analysis (TGA) was investigated by a simultaneous thermal analyzer (STA 449 F3) with a scanning rate of 5 °C min^−1^. Scanning electron microscopy (SEM) and energy dispersion X-ray spectrometer (EDX) were operated by Hitachi S-4300 with the accelerating voltage of 10 kV. The phase composition of SSCN samples was characterized by X-ray diffraction (XRD, D 8, Germany BRUKER-AXS) with Cu-Kα radiation. The X-ray photoelectron spectroscopy (XPS) analysis was operated using an ESCALAB 250 Xi with Mg-Kα radiation.

The electrochemical measurements were manipulated by the RRDE-ALS rotate disk electrode system and CHI 760E electrochemical workstation in three-electrode mode. GCE was used as the working electrode with a diameter of 4 mm, platinum wire as the counter electrode, and Ag/AgCl as the reference electrode. A 0.1 M KOH solution was used as the electrolyte. Before electrochemical tests, oxygen was introduced into the electrolyte solution at least for 30 min to obtain the O_2_-saturation solution. Linear sweep voltammetry (LSV) curves were examined at the rotating rate of 1600 rpm and the scan rate of 5 mV s^−1^. The electron transfer number (*n*) during the ORR process was calculated by the following equation:

$$n=\frac{4\times I_{disk}}{I_{disk}+I_{ring}/N}$$

where $I_{disk}$ is the disk current, and $I_{ring}$ is the ring current. *N* (= 0.4) is the theoretical current collection efficiency of the Pt ring. The electrochemical impedance spectroscopy (EIS) was obtained with 5 mV amplitude within 100 kHz to 0.1 Hz.

## 4. Conclusions

Sm_0.5_Sr_0.5_Co_1__−x_Ni_x_O_3__−δ_ (SSCN, x = 0.2, 0.4, 0.5, 0.6, and 0.8) nanofibers were successfully synthesized by electrospinning method. The synergistic effect of Co and N and the three-dimensional structure of SSCN nanofibers result in a good catalytic activity. In 0.1 M KOH electrolyte solution, SSCN82 with the optimum Co: Ni molar ratio exhibits good ORR/OER catalytic performances and electrochemical double-layer capacitance. The SSCN82 nanofibers are a promising cathode electrocatalyst for metal–air batteries.

## Figures and Tables

**Table 1:** Comparison of OER performances in 0.1 M KOH media for SSCN catalysts with other perovskite catalysts.

Catalysts	OER Onset Potential (E/V vs. RHE)	Tafel Slope (mV dec^−1^)	Current Density at 1.8 V (mA cm^−2^)	References
SSCN82	1.39	123.8	6.01	This work
SSCN64	1.51	129.1	2.2	This work
SSCN55	1.52	133.9	1.4	This work
SSCN46	1.61	138.1	1.12	This work
SSCN28	1.63	159.2	0.65	This work
BSCF900N2		143	ca 7	[15]
SSC-HG	1.53	115		[16]
LSM	1.7	226	2	[36]
LCNP@NCNF	1.51	152	4.09	[37]
LSNF-5546	1.56	76		[38]
IrO_2_	1.56	115	-	[39]

## Data Availability

Data contained within the article and Supplementary Materials are available on request from the authors.

## Sample Availability

Samples of the compounds are not available from the authors.
